# Violent behavior and the network properties of psychopathological symptoms and real-life functioning in patients with schizophrenia

**DOI:** 10.3389/fpsyt.2023.1324911

**Published:** 2024-01-11

**Authors:** Li-Chang Chen, Wen-Yan Tan, Jun-Yan Xi, Xin-Hui Xie, Hai-Cheng Lin, Shi-Bin Wang, Gong-Hua Wu, Yu Liu, Jing Gu, Fu-Jun Jia, Zhi-Cheng Du, Yuan-Tao Hao

**Affiliations:** ^1^Department of Medical Statistics, School of Public Health & Center for Health Information Research & Sun Yat-sen Global Health Institute, Sun Yat-sen University, Guangzhou, Guangdong, China; ^2^Guangdong Mental Health Center, Guangdong Provincial People’s Hospital (Guangdong Academy of Medical Sciences), Southern Medical University, Guangzhou, Guangdong, China; ^3^Department of Psychiatry, Renmin Hospital of Wuhan University, Wuhan, Hubei, China; ^4^School of Public Health and Management, Guangzhou University of Chinese Medicine, Guangzhou, Guangdong, China; ^5^Center for Public Health and Epidemic Preparedness & Response, Peking University, Beijing, China; ^6^Key Laboratory of Epidemiology of Major Diseases, Peking University, Ministry of Education, Beijing, China

**Keywords:** schizophrenia, psychopathological symptoms, real-life functioning, violence, network analysis

## Abstract

**Objective:**

To assess the interplay among psychopathological symptoms and real-life functioning, and to further detect their influence with violent behavior in patient with schizophrenia.

**Methods:**

A sample of 1,664 patients with post-violence assessments and their propensity score–matched controls without violence from a disease registration report system of community mental health service in Guangdong, China, were studied by network analysis. *Ising-Model* was used to estimate networks of psychopathological symptoms and real-life functioning. Then, we tested whether network properties indicated the patterns of interaction were different between cases and controls, and calculated centrality indices of each node to identify the central nodes. Sensitivity analysis was conducted to examine the difference of interaction patterns between pre-violence and post-violence assessments in violence cases.

**Results:**

Some nodes in the same domain were highly positive interrelations, while psychopathological symptoms were negatively related to real-life functioning in all networks. Many symptom-symptom connections and symptom-functioning connections were disconnected after the violence. The network density decreased from 23.53% to 12.42% without statistical significance (*p* = 0.338). The network structure, the global network strength, and the global clustering coefficient decreased significantly after the violence (*p* < 0.001, *p* = 0.019, and *p* = 0.045, respectively). Real-life functioning had a higher node strength. The strength of sleeping, lack of spontaneity and flow of conversation, and preoccupation were decreased in post-violence network of patients.

**Conclusion:**

The decreasing connectivity may indicate an increased risk of violence and early warning for detecting violence. Interventions and improving health state based on nodes with high strength might prevent violence in schizophrenia patients.

## 1 Introduction

While most patients with schizophrenia are never violent, they were found to have higher risk for violence compared with the general population (1–4). A systematic review of 20 studies reported a higher risk of violence in schizophrenia patients compared with the general population, with an odds ratio (OR) of 4.0 in man, and 7.9 in woman (1). Violence can result in not only public safety issue and increasing public health burden, but also negative impact on patients’ health. Over 75% of family caregivers experienced physical violence from their relative with schizophrenia, and schizophrenia patients with violence had longer hospital stay than those without violence (5, 6).

In order to prevent the violence and deliver mental health care for schizophrenia patients, the Chinese government has developed mental health surveillance and integrated mental health into the national primary public health care (7–9). It is along the same lines as the key recommendation of World Health Organization’s Comprehensive Mental Health Action Plan 2013–2030 that provide services in community-based settings (10). Specifically, patients with severe mental illness (schizophrenia, schizoaffective disorder, bipolar disorder, delusional disorder, psychotic disorder due to epilepsy, and intellectual disability) are diagnosed by psychiatrists and provided follow-up services by trained service providers in community health centers. The follow-up mainly includes violent behavior, psychopathological symptoms, real-life functioning, medication, and physical diseases. The risk assessment of violence for patients is based on violent behavior in the last follow-up period (11–13). In other words, patients with high risk of violence are identified based on the history of violent behavior.

To improve the development of risk assessment tools and to identify patients with high risk of violence earlier, it is essential to investigate the risk factors of violence. Among many risk factors that have been identified, a consistent finding is that the relationship between violence and schizophrenia is primarily caused by psychopathological symptoms (1, 14). A meta-analysis of 110 studies reported that violence was more likely to occur in patients with higher total Positive And Negative Symptom Scale scores (PANSS, OR = 1.5), and the psychopathological symptoms was more strongly associated with violence risk (OR = 2.8) in inpatients (14). Moreover, cognitive impairment is one of the core features in people with schizophrenia, and may be associated with violence risk (15–17). However, psychopathological symptoms were insufficient to predict the violent behavior due to its complexity. On the other hand, real-life functioning also plays an important role in violence risk (18–20). For instance, schizophrenia patients with a history of violence had shorter working hours, poorer learning skills and less satisfactory sleep quality than those without violence (20–23). Their disability in real-life functioning resulted in difficulties managing interpersonal conflicts and thus increased the risk of violence (18, 24, 25).

Epidemiological evidence for the influence of psychopathological symptoms and real-life functioning on violence have been identified by analyzing the relationship between violent behavior and risk factors. However, the psychopathological symptoms and real-life functioning interact with each other, and their relationship is complex. For instance, schizophrenia patients are often classified into subgroups with predominantly positive or negative symptoms, and negative and positive symptoms are tightly related to each other (26–29). Symptoms were related to functioning, but the negative symptoms were more strongly associated with lower functional levels than positive symptoms (24, 30, 31). To date, yet the complex interplay between psychopathological symptoms and real-life functioning still remains unclear. As a result, to what extent such interplay could influence violent behavior is unknown, thus slowing down the development of risk assessment tools (32, 33).

In the recent years, network analysis becomes an effective method to investigate complex interactions between multiple variables (32, 34–36). It provides insights into the interplay among different variables by including them as nodes in the same network and connecting to each other by edges that represent their relationships. For example, some researchers conducted a network analysis to examine the interactions among suicide risk factors, which was beneficial to understand the complexity of suicidal behavior (37–39). Other studies applied network analysis to interpret the relationships between psychopathological symptoms and real-life functioning in schizophrenia patients, and negative correlations between specific psychopathological symptoms and real-life functioning were found after accounting for the complex interaction between all nodes (i.e., psychopathological variables, social cognition, social functioning) in the network (40–43). Galderisi et al. found high interconnection in real-life functioning, but less interconnected pattern in psychopathological symptoms in the network included psychopathology, personal resources, context-related factors and real-life functioning in schizophrenia (42).

Moreover, the network analysis provides the network properties (e.g., network structure and global strength) to estimate the overall patterns of relationship among variables. It allows us to investigate the influence of the patterns of relationship by comparing them between groups. Several studies found network properties was associated to some important clinical outcomes (i.e., stronger global strength contributed to maintain symptoms’ activation and disorder persistence) (42, 44–46). In addition, the node centrality, a crucial network characteristic to measure the importance of each node within the network, could suggest potential valuable targets for the prediction and prevention. For example, Gijzen et al. found loneliness and sadness were the most central variables in the depression symptoms network with a relatively high central index strength and expected influence (47). Hu et al. reported that the motivation and pleasure could be served as the potential intervention targets to improve social functioning in schizophrenia patients because of its high strength centrality among negative symptoms, social functioning and other psychopathology (43).

In light of the advantages in terms of interpreting relationships among a large number of variables, network analysis might be consequently employed to address the complex interplay between psychopathological symptoms and real-life functioning, and thus to provide new insights of their influence on violence under real-world setting. However, yet few studies have been focused on this aspect, to the best of our knowledge. Here, we aimed to examine the interdependency patterns among risk factors, and to further detect their connection and influence with violent behavior. Additionally, the target risk factors to provide intervention evidence were also identified. Based on earlier studies (40–42, 47, 48), we hypothesized that there is a weaker interconnected network after violent behavior, with real-life functioning play a more crucial role rather than psychopathological symptoms in the network.

## 2 Methods

### 2.1 Participants

Data for this network analysis were drawn from Guangdong Mental Health Center Network Medical System (GDMHS), a disease registration report system of community mental health service in Guangdong, China. This system was established to report the epidemiological characteristics and follow-up health records of six categories of severe mental illnesses defined by the National Health Commission of the People’s Republic of China, including schizophrenia, schizoaffective disorder, bipolar disorder, delusional disorder, psychotic disorder due to epilepsy, and intellectual disability (9, 11). Primarily, the system engaged patients who were referred from health institutes or screened quarterly by local Community Health Service Centers as required by the government’s health laws and regulations (7). Further confirmation of diagnosis for patients with severe mental illnesses was conducted by psychiatrists. Then, public health services were provided to patients by the regular follow-ups by the community health care staff (7, 49). Patients were followed at least once every 3 months. In each follow-up, the community health care staff should evaluate patients’ disease condition (e.g., violent behaviors, self-harm, psychopathological symptoms, real-life functioning, adverse drug reactions, serious physiological diseases). In addition, they were responsible for providing medication prescriptions and guidance on treatment and rehabilitation.

We included schizophrenia patients with the International Classification of Diseases, Tenth Revision code F20* in GDMHS. Patients with at least one physical violence against others during the period from January 1, 2019, to December 31, 2020 were identified as violence cases. Another inclusion criterion in violence cases was at least six-month follow-up before their first follow-up assessment after the first violent behavior. Patients without any record of violence between January 1, 2019, and December 31, 2020, were identified as controls. General exclusion criteria were: (1) present or history of severe neurological diseases (e.g., neurodegenerative diseases, cerebrovascular diseases, neurological tumors, neurological infectious diseases); (2) with psychopathological comorbidities, including intellectual disability, major depressive disorder, and bipolar disorder; and (3) presence or history of alcohol or substance abuse.

The research ethics committee of the Guangdong Mental Health Center in China approved the study (authorization No.GDMHR2019201H) on 13th Oct. 2019.

### 2.2 Assessment

Psychopathological symptoms and real-life functioning were evaluated following *Instructions on the Assessment of Psychopathological Symptoms and Real-life Functioning in Patients with Severe Mental Illnesses* published by the Chinese Center for Disease Control and Prevention (see Supplementary Table S1). The PANSS, Modified Overt Aggression Scale (MOAS), and Specific Level of Functioning Scale (SLOF) were referenced in the development of instructions. Psychopathological symptoms include eleven binary-coded (Present symptom =1, Absent/Questionable pathology =0) symptoms: hallucinatory behavior, suspiciousness and persecution, excitement, lack of spontaneity and flow of conversation, passive apathetic social withdrawal, mannerisms and posturing, depression, wandering, preoccupation, affective lability, aggression. Real-life functioning were also binary data (Totally self-sufficient = 1, Needs help or totally dependent = 0) and includes seven items: sleeping, eating, personal care skills, household management, work skills, study skills, interpersonal relationships. Community public health service workers or family doctors in community health clinics were responsible for the assessment based on the instructions after appropriate training.

### 2.3 Statistical analysis

Propensity score matching was completed based on age, sex, year of services delivered, and history of violence against self or others to select 2 controls per violent case. We used data collected from violence cases at the first follow-up assessment after their first violence, and randomly selected one follow-up assessment from controls.

Demographics, clinical characteristics, symptoms, and functioning were described using means and standard deviations (SD) for continuous variables and as frequencies and percentages for categorical variables. Two-sample *t*-test and Chi-square test were used to examine differences in clinical characteristics, symptoms and functioning between violence cases and controls, respectively. A phi coefficient was calculated to measure the strength of association between each pairwise combination of psychopathological symptoms and real-life functioning.

Psychopathological symptoms and real-life functioning were included as nodes in network analysis. We used the *Ising-Model* to estimate the networks for cases and controls, separately (50). The network represents the correlations between all pairs of nodes after controlling the influence of all the variables. To control spurious correlations, we used Least Absolute Shrinkage and Selector Operator (LASSO) regularization with the Extended Bayesian Information Criterion (EBIC) model selection. A hyperparameter γ is used to control the sparsity of the network in EBIC. Lower γ values lead to models with more edges and higher sensitivity. We set the hyperparameter γ of EBIC to 0.5 (51, 52). To explore the influence of the hyperparameter γ to the networks, we computed the EBIC based on γ from 0 to 0.5 with 0.1-intervals, and the result show that the higher hyperparameter γ indicated fewer edges but varying γ changed the results slightly (see Supplementary Figure S1 and Supplementary Table S2).

In this study, we focused on five network properties (53) and three centrality indices (54, 55). Details regarding them are summarized in Table 1. Differences in network properties were assessed using resampling-based permutation testing (with 1,000 permutations) (56). For each centrality indices, z-transformation was performed to facilitate comparisons. A Higher value indicates the higher centrality of a node. In addition, we assessed the stability of the centrality indices by the case-dropping bootstrap method (with 1,000 samples). The correlation stability coefficient (CS-coefficient) was used to evaluate the stability of centrality estimates. The CS-coefficient indicates the maximum percentage of samples that can be dropped to preserve 95% probability a correlation of at least 0.7 between original centrality indices and subsamples’ centrality indices. Epskamp et.al recommended the CS-coefficient should not be lower than 0.25, and preferably above 0.5 (57).

**Table 1:** Summary of the network properties and centrality indices.

Measure	Metrics	Definition	Practical meaning
Network properties	Network structure	Distribution of edge weights	How the level of connectivity of each node is distributed in the network
	Global network strength	The weighted sum of the absolute connections	Level of connectivity of the network
	Network density	The fraction of edges presents over all possible edges	To what extent nodes in the network are interconnected
	Global clustering coefficient, CC	The ratio of the number of closed triplets to the number of paths of length two in network	To what extent nodes in the network tend to cluster together
	Global average shortest path length, ASPL	Average length along the shortest weighted path lengths for all possible pairs of network nodes	Level of information efficiency in the network
Centrality indices	Strength	The weighted sum of the absolute connections of one node to all other nodes	Level of connectivity of a node in the network
	Closeness	The inverse of the weighted sum of shortest path lengths from one node to all other nodes	Level of speed of a node connected to other nodes
	Betweenness	The frequency of one node lies on all the shortest path length between other nodes	Degree to which the node acts as a bridge between other nodes

All statistical analyses were conducted using R version 4.1.0. Networks were created and visualized using the R-package *bootnet* and *qgraph* (57, 58). The R-package *bootnet* and *qgraph* were also used to estimate centrality indices and assess network stability (56, 59).The R-package *NetworkComparisonTest* and *NetworkToolbox* were used to test the difference in network properties among networks (56, 59).

To provide additional insights into the understanding of the pattern of the complex interplay among variables, network analysis was performed in pre-violence and post-violence assessments in case group. We used data collected from violence cases at the first follow-up assessment after their first violence (T), three-month (T-3), and six-month(T-6) follow-up assessments before their first violence to compare differences in network properties and centrality indices before (3 and 6 months before: T-3 and T-6) and after (T) the violence.

## 3 Results

There were 342,806 schizophrenia patients with at least one follow-up in the study period (January 1, 2019 to December 31, 2020), and 4,860 of them had at least one violent behavior record. A total of 1,667 patients had at least a six-month follow-up before their first follow-up assessment after the first violence. Propensity score matching with 1:2 ratio resulted in 1664 violence cases and 3,327 controls in the present study.

### 3.1 Sample characteristics

Violence cases were predominantly observed in males (67.1%), with a mean age of 43.8 years (SD = 13.4). In violence cases, the mean onset age was 27.8 years (SD = 12.0), and the mean duration of illness was 16.1 years (SD = 10.5). Among two groups, 61.4% had history of violence against self and others. Both groups were well balanced in clinical characteristics. Incidences of psychopathological symptoms in violence cases were significantly higher than controls, except for the incidence of passive apathetic social withdrawal. Similarly, all real-life functioning significantly deteriorated in violence cases compared with that in controls. The demographic and clinical characteristics, and network variables were shown in Table 2.

**Table 2:** Demographic and clinical characteristics, and network variables in violence cases and controls.

Characteristics	Violence cases (*N* = 1,664)	Propensity score-matched controls (*N* = 3,327)	*p*
Sex			
Male, *n* (%)	1,116 (67.1)	2,232 (67.1)	–
Female, *n* (%)	548 (32.9)	1,095 (32.9)	–
Age (years), mean ± SD	43.8 ± 13.4	43.2 ± 13.4	–
Age at onset (years), mean ± SD	27.8 ± 12.0	27.9 ± 12.3	0.711
Duration of illness (years), mean ± SD	16.1 ± 10.5	15.8 ± 10.6	0.346
History of violence against self and others, *n* (%)	1,022 (61.4)	2044 (61.4)	–
Hallucinatory behavior, *n* (%)	354 (21.3)	85 (2.6)	<0.001
Suspiciousness and persecution, *n* (%)	347 (20.9)	141 (4.2)	<0.001
Excitement, *n* (%)	215 (12.9)	108 (3.2)	<0.001
Lack of spontaneity and flow of conversation, *n* (%)	396 (23.8)	308 (9.3)	<0.001
Passive apathetic social withdrawal, *n* (%)	211 (12.7)	356 (10.7)	0.042
Mannerisms and posturing, *n* (%)	665 (40.0)	317 (9.5)	<0.001
Depression, *n* (%)	31 (1.9)	12 (0.4)	<0.001
Wandering, *n* (%)	161 (9.7)	46 (1.4)	<0.001
Preoccupation, *n* (%)	260 (15.6)	169 (5.1)	<0.001
Affective lability, *n* (%)	681 (40.9)	155 (4.7)	<0.001
Aggression, *n* (%)	825 (49.6)	19 (0.6)	<0.001
Sleeping, *n* (%)	170 (10.2)	1795 (54.0)	<0.001
Eating, *n* (%)	335 (20.1)	1917 (57.6)	<0.001
Personal care skills, *n* (%)	183 (11.0)	1,450 (43.6)	<0.001
Household management, *n* (%)	83 (5.0)	1,098 (33.0)	<0.001
Work skills, *n* (%)	35 (2.1)	722 (21.7)	<0.001
Study skills, *n* (%)	49 (2.9)	636 (19.1)	<0.001
Interpersonal relationships, *n* (%)	27 (1.6)	661 (19.9)	<0.001

Sex, age, and history of violence against self and others were used to perform propensity score matching.

The phi coefficient between each pairwise combination of psychopathological symptoms and real-life functioning in each group was positive. However, the correlation between psychopathological symptoms and the correlation between psychopathological symptoms and real-life functioning were very low (i.e., phi coefficient < 0.3). Relatively high positive relationship (e.g., phi coefficient > 0.5) were found between real-life functioning. The correlation matrices are shown in Supplementary Figure S2.

### 3.2 Network estimation

**Figure 1:**
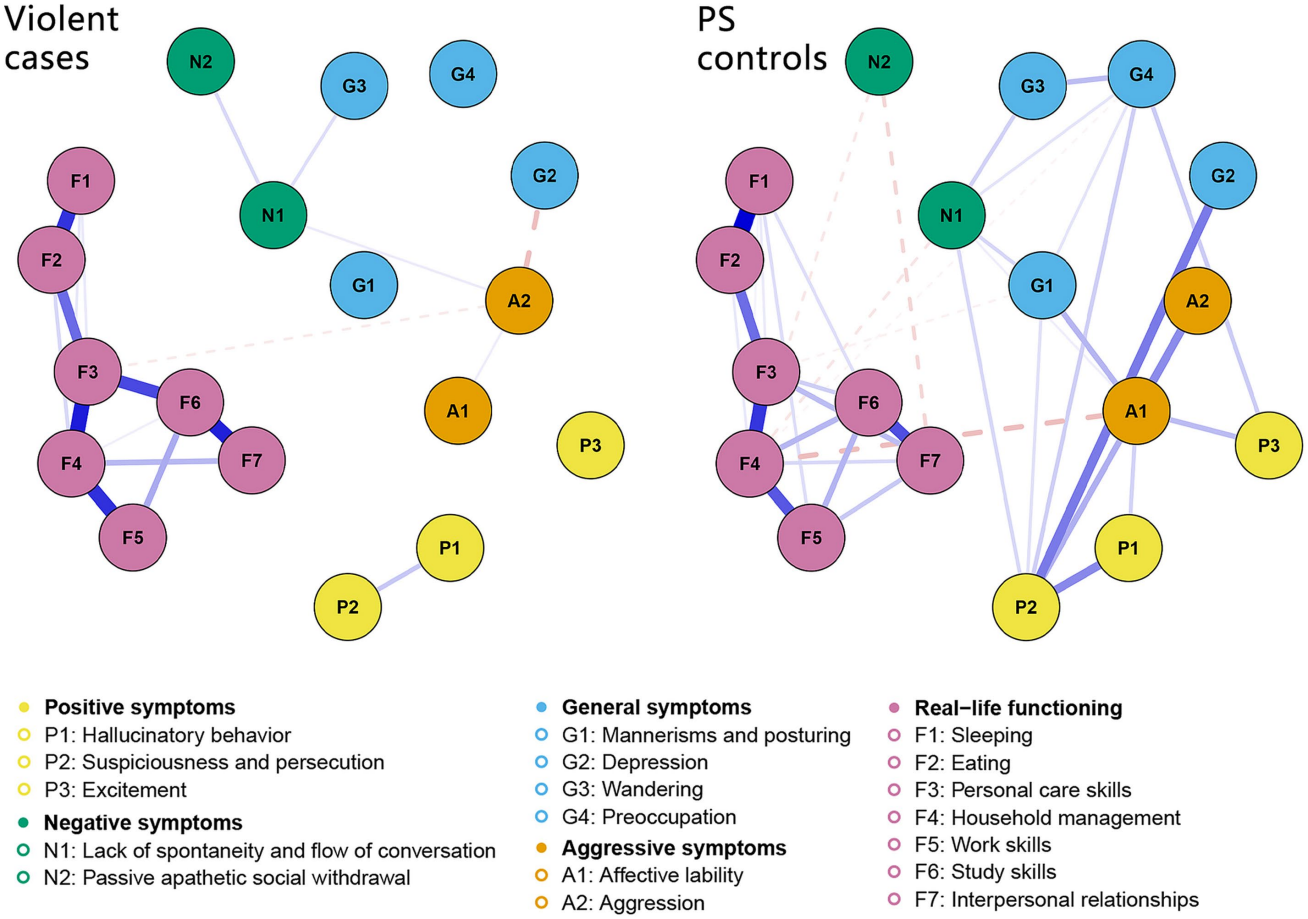
Networks of Psychopathological symptoms and real-life functioning. Solid edges indicate positive relationships, and dashed edges are negative relationships. The thickness of an edge represents the magnitude of the relationship. PS, propensity score–matched.

Figure 1 shows the networks in two groups. Visual inspection showed several similarities between two networks. For example, there were highly positive interrelations between real-life functioning, and some positive connections between psychopathological symptoms (i.e., hallucinatory behavior with suspiciousness and persecution, affective lability with aggression). In addition, some negative connections between psychopathological symptoms and real-life functioning were also identified. On the other hand, there were also pronounced differences between the control network and the post-violence network. Psychopathological symptoms were less interconnected after violence compared to the control network. More specifically, a large number of links between psychopathological symptoms and real-life functioning (i.e., personal care skills with mannerisms and posturing, household management with affective lability) disappeared while some negative connections (Aggression with depression, aggression with personal care skills) emerged in the post-network. Adjacency matrixes (including value of edge weights of adjacent nodes) of networks can be found in Supplementary Table S3.

### 3.3 Network properties

Table 3 shows network properties and comparisons between two groups. The network structure was a significant difference between the violence cases and controls (*p* < 0.001), which means the differences in the distribution of edge weights were significant. The global network strength and the global clustering coefficient significantly decreased in the violence cases (*p* = 0.019 and *p* = 0.045, respectively). The network density and the global average shortest path length decreased in the violence cases, but not significantly.

**Table 3:** Network properties and comparisons in violence cases and propensity score–matched controls.

Network properties	Violence cases	Propensity score–matched controls	Propensity score–matched controls vs violence cases	
			Test statistic	*p*
Network structure	NA	NA	2.151	<0.001
Global network strength	25.665	42.234	16.568	0.019
Network density	12.42%	23.53%	11.11%	0.338
CC	0.294	0.539	0.245	0.045
ASPL	1.561	2.111	0.550	0.016

NA, not applicable; CC, global clustering coefficient, ASPL, global average shortest path length.

### 3.4 Network centrality

**Figure 2:**
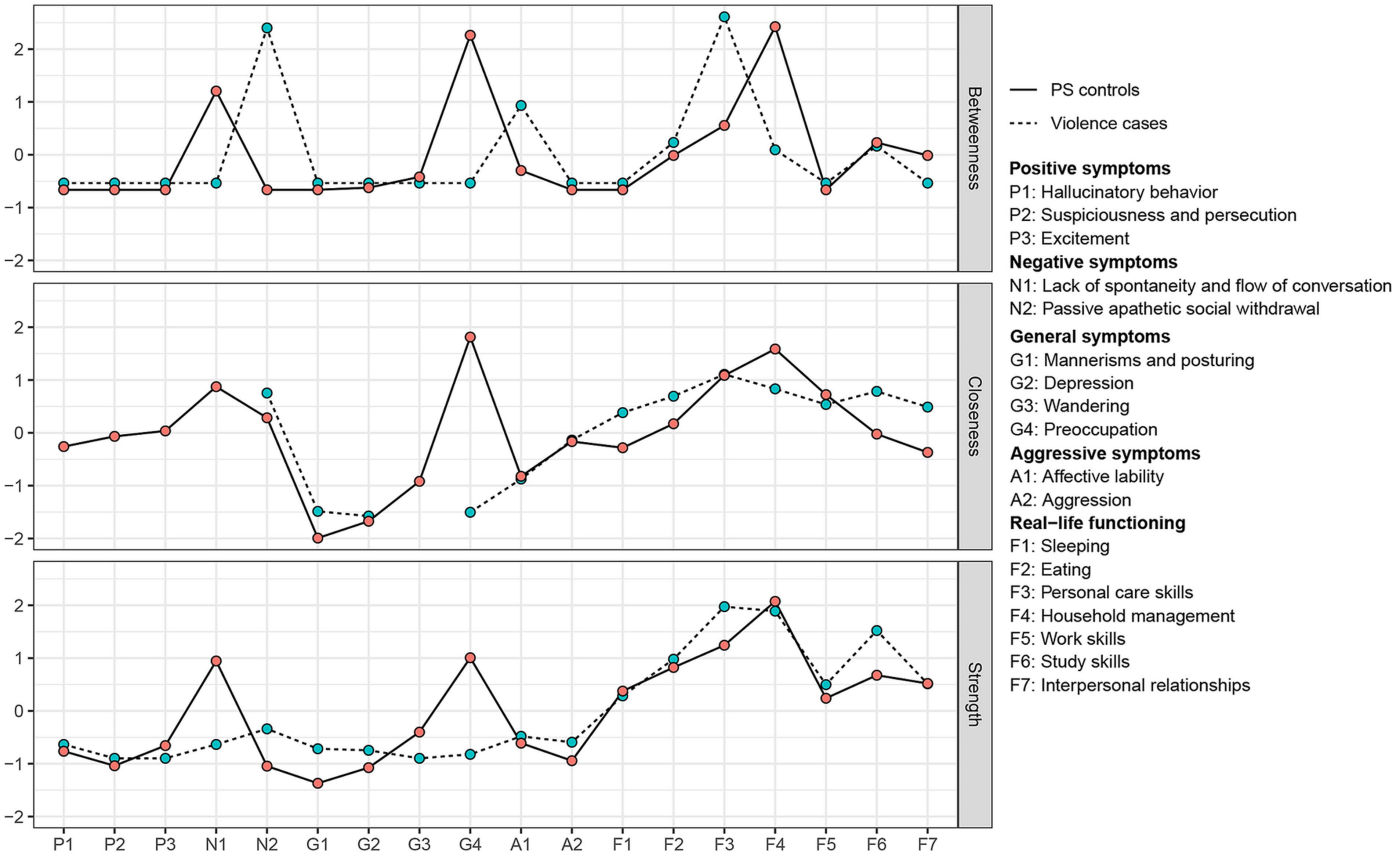
The standardized centrality measures of networks. Closeness is missing because some nodes are isolate in post-violence network. PS, propensity score–matched.

In terms of centrality measures, we only compared strength between two groups because of their reliable estimations. The CS-coefficients for strength were 0.75 in violence cases and controls, while the CS-coefficients for betweenness and closeness were below 0.05 in both groups. The results of the stability analysis of centrality indices are shown in Table 4, and other similar results for different γ values are shown in Supplementary Table S4. Figure 2 shows the centrality measures in two groups. Nodes belonging to real-life functioning had higher strengths in most cases. Sleeping and household management had a lower strength in violence cases. Concerning psychopathological symptoms, excitement, lack of spontaneity and flow of conversation, wandering and preoccupation had lower strength in the post-violence network.

**Table 4:** The correlation stability coefficients for centrality indices.

Group	Strength	Betweenness	Closeness
Violence cases	0.750	0	0
Propensity score–matched controls	0.750	0.050	0

### 3.5 Sensitivity analysis

In the sensitivity analysis, 1,667 violence cases with pre-violence and post-violence follow-up assessments were included. The results of the network comparison between pre-violence and post-violence were very similar to those reported above. However, differences in the network structure were not statistically significant while network density were significantly decreased after violence. For more detailed information on the sensitivity analysis, see Supplementary Tables S5–S8 and Supplementary Figures S3, S4.

## 4 Discussion

Our study examined the pattern of relationships between psychopathological symptoms and real-life functioning in patients with schizophrenia using real-world data, suggesting that post-violence network was weaker connected than the PS controls. Real-life functioning played a critical role in influencing global network strengths, and some symptoms or functioning featured to decrease in strength in the post-violence network.

Several connections presented unanimously in violence cases and their PS controls. Positive interconnections of some symptoms, especially those in the same domain (i.e., hallucinatory behavior with suspiciousness and persecution, affective lability with aggression), were stable regardless of the period of violence and the occurrence of violent behavior. It suggested these symptoms had reliable patterns of co-activation. For instance, aggression was likely to occur when affective lability was occurred, while others were not. These results were consistent with the previous studies, in which the symptoms in the same domain were more related with each other (60). Realizing the co-activation of symptoms may improve the efficacy of assessment provided by community health care staff. They can pay more attention on the occurrence of these co-activation symptoms during the assessment, thereby reducing recall bias. Similarly, real-life functioning was likely to have a strong positive relationship between each other, suggesting they can influence each other to a greater degree. From a network perspective, the tightly connected real-life functioning features implied another opportunity, namely improving one or some functioning may contribute to functional recovery comprehensively. In addition, psychopathological symptoms were negatively related to real-life functioning, being consistent with previous network analysis studies (41–43, 61). Our findings of the pattern of interaction in psychopathological symptoms as well as in real-life functioning were consistent with the network theory of mental disorders, in which heterogeneous nature of psychopathology and causal interactions are present (33, 62).

We found that the network was less interconnected after violence. The number of symptom-symptom connections was decreased after violence. Some functioning- symptom connections (i.e., personal care skills with mannerisms and posturing, household management with affective lability) were vanished in the network of schizophrenia patients after violence. We may hypothesize that the disappearance of these connections indicates the deteriorated real-life functioning as well as psychopathological symptoms. A similar finding was also reported in the study by Galderisi S et al., who found that the emergence of direct connections between cognition variables and real-life functioning domains in subjects with schizophrenia reflected the slight improvements observed in social cognition and neurocognition variables (42). Moreover, the frequency increase was larger in positive and aggressive symptoms compared with other symptoms, which may make relatively weak connections disappear but maintain the interactions that are strong enough (14, 33, 62). Those disappearance of connections reflected the complex dynamics of the interplay of risk factors. However, verifying these hypotheses is out the scope of in this study, and further exploration will be carried out in the future study.

The sparser network and weaker connections between psychopathological symptoms and real-life functioning after the violence contributed to the significant decline of some network properties, suggesting decreased network connectivity and increased violence risk. The global network strengths decreased significantly after violent behavior. Although significant differences were not always found in network structures and network density, we can see the descending trend after violence. Weaker global network strengths in the post-violence network suggests that less connectivity of interplay among risk factors increased the risk for violence. In other words, our finding indicates that a weaker connected pattern of psychopathological symptoms and real-life functioning may predict a higher risk of violence. Then, we could develop risk measurement tools to predict violence using this network property (63, 64). Moreover, network interventions, changing the network strengths, maybe another way to prevent violence in schizophrenia patients (33, 35, 65, 66). For example, community rehabilitation for improving real-life functioning and timely intervention (i.e., modification of treatment regimens, a referral to a psychiatric hospital, strengthened follow-up) of deteriorated symptoms may decrease the risk of violence in patients with schizophrenia (7, 67).

We found that the variables of real-life functioning showed high values of strength centrality, indicating important roles in network. Moreover, the decrease of strengths for some nodes (i.e., sleeping, lack of spontaneity and flow of conversation, preoccupation) may result in weaker global network strengths and higher risk of violence. The stage of these nodes might have relatively great impact on network properties and thus occurrence of violence. The assessment of these nodes needs more concern by community health care staff during the follow-up period. Furthermore, changing the stage of these variables may prevent the decline of global network strengths, as well as the risk of violence. Therefore, these nodes may be a potential intervention target to decrease the risk of violence in schizophrenia patients. Since concentration networks in our study were weakly connected (i.e., having some unconnected variable) and had multiple shortest paths connecting real-life functioning and psychopathological symptoms, closeness and betweenness centrality indices cannot provide more evidence about node importance. However, the previous studies suggested that strength was the most suitable parameter in psychological networks and had the highest prediction value (35, 68–70).

This study holds significant clinical implications for the treatment and management of patients with schizophrenia. Firstly, clinicians can leverage the decreased connection between psychopathological symptoms and functioning to identify patients at a higher risk of violence. Our finding suggested that a reduction in connectivity may indicate an increased likelihood of violent behavior. For example, the negative relationships between psychopathological symptoms and functioning were diminished. Secondly, our study highlighted the importance of real-life functioning within the network, aligning with recovery-oriented approaches to schizophrenia (41, 71). This emphasizes achieving a meaningful life for patients beyond symptom management. In other words, treatments beyond antipsychotic drugs are essential to decrease the risk of violence. Finally, the identification of central symptoms, including sleeping, lack of spontaneity and flow of conversation, and preoccupation, as potential intervention targets can enhance efficiency in routine clinical practice, especially in resource-constrained settings.

Strengths of this study are the relatively large sample size to ensure a stable network estimation (57). We included both the real-life functioning and psychopathological symptoms under the real-world setting to explain the complexity of violent behavior via network analysis.

However, there are some limitations in this study. First, we used self-developed questionnaire instead of scales to measure psychopathological symptoms and real-life functioning in this study. It is infeasible to implement scales in the present study because there are lack of workforce and training for raters in community health clinics. To reduce measure bias and improve the feasibility in the real-world setting, the self-developed questionnaire with simple instruction and direction were used. Future research should implement scales to replicate the network structure. Second, we did not include insight and cognitive impairment to our network because there is lack of suitable assessment tool and trained mental health care staff in community setting. We included psychopathological symptoms and real-life functioning, which can be predicted by insight or cognitive impairment and be easier to evaluate and intervene (72, 73). In future studies, feasible measurements of insight and cognitive impairment should be considered to enhance our understanding of the occurrence of violence. Finally, our between-subjects design may preclude the representability of our present network properties to individuals. Longitudinal studies (i.e., experience sampling) are required to examine how the change in the complex interplay between psychopathological symptoms and real-life functioning would influence violent behavior at the level of the individual patient.

In conclusion, our study advances our knowledge on complex interplay among psychopathological symptoms and real-life functioning, and their influence on violent behavior in patient with schizophrenia under the real-world setting. Many connections were stable while some were disconnected after violence. Both the number and strengths of the connections decreased after violence, which results in significant decrease in network connectivity (i.e., network structure, global network strength). Further, real-life functioning had relatively high strength centrality, and strengths for some variables (i.e., sleeping, lack of spontaneity and flow of conversation, preoccupation) decreased after violence. The changes in network connectivity and centrality indices indicated a promising line of the development of risk assessment tools. Further, our findings highlighted the potential of real-life functioning, mannerisms and posturing, and affective lability as the intervention target for preventing violence.

## Data availability statement

The data analyzed in this study is subject to the following licenses/restrictions: The data that support the findings of this study are available from Guangdong Mental Health Center but restrictions apply to the availability of these data, which were used under license for the current study, and so are not publicly available. Data are however available from the authors upon reasonable request and with permission of Guangdong Mental Health Center. Requests to access these datasets should be directed to jiafujun@126.com.

## Ethics statement

The studies involving humans were approved by the research ethics committee of the Guangdong Mental Health Center in China (authorization No.GDMHR2019201H). The studies were conducted in accordance with the local legislation and institutional requirements. Written informed consent for participation was not required from the participants or the participants’ legal guardians/next of kin in accordance with the national legislation and institutional requirements.

## Author contributions

L-CC: Conceptualization, Writing – review & editing, Formal analysis, Methodology, Visualization, Writing – original draft. W-YT: Conceptualization, Formal analysis, Visualization, Writing – original draft, Writing – review & editing. J-YX: Writing – original draft, Writing – review & editing. X-HX: Conceptualization, Methodology, Validation, Writing – review & editing. H-CL: Writing – review & editing. S-BW: Methodology, Writing – review & editing. G-HW: Methodology, Validation, Writing – review & editing. YL: Writing – review & editing. JG: Writing – review & editing. F-JJ: Supervision, Writing – review & editing. Z-CD: Conceptualization, Supervision, Writing – original draft, Writing – review & editing. Y-TH: Funding acquisition, Supervision, Writing – review & editing.

## References

[1] Fazel S Gulati G Linsell L Geddes JR Grann M Mcgrath JJPM. Schizophrenia and violence: systematic review and Meta-analysis. PLoS Med. (2009) 6:e1000120. doi: 10.1371/journal.pmed.1000120, PMID: 19668362 PMC2718581

[2] Ouzir M. Impulsivity in schizophrenia: a comprehensive update. Aggress Violent Behav. (2013) 18:247–54. doi: 10.1016/j.avb.2012.11.014, PMID: 22212849

[3] Fazel S Wolf A Palm C Lichtenstein PJTLP. Violent crime, suicide, and premature mortality in patients with schizophrenia and related disorders: a 38-year Total population study in Sweden. Lancet Psychiatry. (2014) 1:44–54. doi: 10.1016/S2215-0366(14)70223-8 25110636 PMC4124855

[4] Whiting D Lichtenstein P Fazel S. Violence and mental disorders: a structured review of associations by individual diagnoses, risk factors, and risk assessment. Lancet Psychiatry. (2021) 8:150–61. doi: 10.1016/S2215-0366(20)30262-5, PMID: 33096045

[5] Kageyama M Solomon P Yokoyama K Nakamura Y Kobayashi S Fujii C. Violence towards family caregivers by their relative with schizophrenia in Japan. Psychiatry Q. (2018) 89:329–40. doi: 10.1007/s11126-017-9537-4, PMID: 28971267

[6] Girasek H Nagy VA Fekete S Ungvari GS Gazdag G. Prevalence and correlates of aggressive behavior in psychiatric inpatient populations. World J Psychiatry. (2022) 12:1–23. doi: 10.5498/wjp.v12.i1.1, PMID: 35111577 PMC8783168

[7] The National Health Commission of the People's Republic of China. Practices of treatment and Management of Severe Mental Illness. Available at: http://www.gov.cn/gongbao/content/2018/content_5338247.htm?ivk_sa=1024320u(2018) (Accessed October 20, 2022).

[8] The National Health Commission of the People's Republic of China. Specification of National Basic Public Health Services. Available at: http://www.nhc.gov.cn/ewebeditor/uploadfile/2017/04/20170417104506514.pdf (Accessed October 20, 2022).

[9] Liang D Mays VM Hwang WC. Integrated mental health Services in China: challenges and planning for the future. Health Policy Plan. (2018) 33:107–22. doi: 10.1093/heapol/czx137, PMID: 29040516 PMC5886187

[10] World Health Organization. Comprehensive mental health action plan 2013–2030. Available at: https://www.who.int/publications/i/item/9789240031029 (Accessed October 20, 2022).

[11] Zhou W Xiao S. Reporting on China's mental health surveillance. Am J Psychiatry. (2015) 172:314–5. doi: 10.1176/appi.ajp.2015.14070945, PMID: 25827029

[12] Liu Y Liu X Wen H Wang D Yang X Tang W. Risk behavior in patients with severe mental disorders: a prospective study of 121,830 patients managed in rural households of Western China. BMC Psychiatry. (2018) 18:134. doi: 10.1186/s12888-018-1709-8 29776345 PMC5960100

[13] Tan W Lin H Lei B Ou A He Z Yang N. The psychosis analysis in real-world on a cohort of large-scale patients with schizophrenia. BMC Med Inform Decis Mak. (2020) 20:132. doi: 10.1186/s12911-020-1125-0, PMID: 32646484 PMC7477870

[14] Witt K van Dorn R Fazel S. Risk factors for violence in psychosis: systematic review and Meta-regression analysis of 110 studies. PLoS One. (2013) 8:e55942. doi: 10.1371/journal.pone.0055942, PMID: 23418482 PMC3572179

[15] Vita A Gaebel W Mucci A Sachs G Barlati S Giordano GM. European psychiatric association guidance on treatment of cognitive impairment in schizophrenia. Eur Psychiatry. (2022) 65:e57. doi: 10.1192/j.eurpsy.2022.2315, PMID: 36059103 PMC9532218

[16] Reinharth J Reynolds G Dill C Serper M. Cognitive predictors of violence in schizophrenia: a meta-analytic review. Schizophr Res Cogn. (2014) 1:101–11. doi: 10.1016/j.scog.2014.06.001

[17] Barlati S Nibbio G Stanga V Giovannoli G Calzavara-Pinton I Necchini N. Cognitive and clinical characteristics of offenders and non-offenders diagnosed with schizophrenia Spectrum disorders: results of the Recoviwel observational study. Eur Arch Psychiatry Clin Neurosci. (2023) 273:1307–16. doi: 10.1007/s00406-022-01510-9, PMID: 36309882

[18] Ahmed AO Richardson J Buckner A Romanoff S Feder M Oragunye N. Do cognitive deficits predict negative emotionality and aggression in schizophrenia? Psychiatry Res. (2018) 259:350–7. doi: 10.1016/j.psychres.2017.11.003, PMID: 29120842

[19] Moulin V Palix J Golay P Dumais A Gholamrezaee MM Azzola A. Violent behaviour in early psychosis patients: can we identify clinical risk profiles? Early Interv Psychiatry. (2019) 13:517–24. doi: 10.1111/eip.12512, PMID: 29143486

[20] Kashiwagi H Matsumoto J Miura K Takeda K Yamada Y Fujimoto M. Neurocognitive features, personality traits, and social function in patients with schizophrenia with a history of violence. J Psychiatr Res. (2022) 147:50–8. doi: 10.1016/j.jpsychires.2022.01.012, PMID: 35021134

[21] Schug RA Raine A. Comparative Meta-analyses of neuropsychological functioning in antisocial schizophrenic persons. Clin Psychol Rev. (2009) 29:230–42. doi: 10.1016/j.cpr.2009.01.004, PMID: 19278761

[22] Engelstad KN Vaskinn A Torgalsbøen A-K Mohn C Lau B Rund BR. Impaired neuropsychological profile in homicide offenders with schizophrenia. Compr Psychiatry. (2018) 85:55–60. doi: 10.1016/j.comppsych.2018.06.002, PMID: 29981505

[23] Chen ZT Wang HT Chueh KH Liu IC Yang CM. An exploration of the sleep quality and potential violence among patients with schizophrenia in community. Perspect Psychiatr Care. (2021) 57:648–54. doi: 10.1111/ppc.12589, PMID: 32730660

[24] O'Reilly K Donohoe G Coyle C O'Sullivan D Rowe A Losty M. Prospective cohort study of the relationship between neuro-cognition, social cognition and violence in forensic patients with schizophrenia and schizoaffective disorder. BMC Psychiatry. (2015) 15:155. doi: 10.1186/s12888-015-0548-0 26159728 PMC4496853

[25] Galderisi S Rossi A Rocca P Bertolino A Mucci A Bucci P. The influence of illness-related variables, personal resources and context-related factors on real-life functioning of people with schizophrenia. World Psychiatry. (2014) 13:275–87. doi: 10.1002/wps.20167, PMID: 25273301 PMC4219069

[26] Carrà G Crocamo C Angermeyer M Brugha T Toumi M Bebbington P. Positive and negative symptoms in schizophrenia: a longitudinal analysis using latent variable structural equation modelling. Schizophr Res. (2019) 204:58–64. doi: 10.1016/j.schres.2018.08.018, PMID: 30177344

[27] Vanes LD Mouchlianitis E Patel K Barry E Wong K Thomas M. Neural correlates of positive and negative symptoms through the illness course: an Fmri study in early psychosis and chronic schizophrenia. Sci Rep. (2019) 9:14444. doi: 10.1038/s41598-019-51023-0 31595009 PMC6783468

[28] Jauhar S Johnstone M McKenna PJ. Schizophrenia. Lancet. (2022) 399:473–86. doi: 10.1016/S0140-6736(21)01730-X, PMID: 35093231

[29] Kay SR Fiszbein A Opler LA. The positive and negative syndrome scale (Panss) for schizophrenia. Schizophr Bull. (1987) 13:261–76. doi: 10.1093/schbul/13.2.261 3616518

[30] Rabinowitz J Levine SZ Garibaldi G Bugarski-Kirola D Berardo CG Kapur S. Negative symptoms have greater impact on functioning than positive symptoms in schizophrenia: analysis of Catie data. Schizophr Res. (2012) 137:147–50. doi: 10.1016/j.schres.2012.01.015, PMID: 22316568

[31] Galderisi S Bucci P Mucci A Kirkpatrick B Pini S Rossi A. Categorical and dimensional approaches to negative symptoms of schizophrenia: focus on long-term stability and functional outcome. Schizophr Res. (2013) 147:157–62. doi: 10.1016/j.schres.2013.03.020, PMID: 23608244

[32] de Beurs D Bockting C Kerkhof A Scheepers F O’Connor R Penninx B. A network perspective on suicidal behavior: understanding suicidality as a complex system. Suicide Life Threat Behav. (2021) 51:115–26. doi: 10.1111/sltb.12676, PMID: 33624872 PMC7986393

[33] Borsboom D. A network theory of mental disorders. World Psychiatry. (2017) 16:5–13. doi: 10.1002/wps.20375, PMID: 28127906 PMC5269502

[34] Borsboom D Cramer AO. Network analysis: an integrative approach to the structure of psychopathology. Annu Rev Clin Psychol. (2013) 9:91–121. doi: 10.1146/annurev-clinpsy-050212-185608, PMID: 23537483

[35] Robinaugh DJ Hoekstra RHA Toner ER Borsboom D. The network approach to psychopathology: a review of the literature 2008–2018 and an agenda for future research. Psychol Med. (2020) 50:353–66. doi: 10.1017/S0033291719003404, PMID: 31875792 PMC7334828

[36] Ruan Q-N Chen C-M Yang J-S Yan W-J Huang Z-X. Network analysis of emotion regulation and reactivity in adolescents: identifying central components and implications for anxiety and depression interventions. Front Psych. (2023) 14:1230807. doi: 10.3389/fpsyt.2023.1230807, PMID: 37867768 PMC10586221

[37] Graziano RC Aunon FM LoSavio ST Elbogen EB Beckham JC Workgroup VAM-AM. A network analysis of risk factors for suicide in Iraq/Afghanistan-era veterans. J Psychiatr Res. (2021) 138:264–71. doi: 10.1016/j.jpsychires.2021.03.065, PMID: 33872963 PMC8192445

[38] Núñez D Ulloa JL Guillaume S Olié E Alacreu-Crespo A Courtet P. Suicidal ideation and affect lability in single and multiple suicidal attempters with major depressive disorder: an exploratory network analysis. J Affect Disord. (2020) 272:371–9. doi: 10.1016/j.jad.2020.04.004, PMID: 32553380

[39] De Beurs D Fried EI Wetherall K Cleare S Db OC Ferguson E. Exploring the psychology of suicidal ideation: a theory driven network analysis. Behav Res Ther. (2019) 120:103419. doi: 10.1016/j.brat.2019.103419, PMID: 31238299

[40] Hajduk M Penn DL Harvey PD Pinkham AE. Social cognition, Neurocognition, symptomatology, functional competences and outcomes in people with schizophrenia – a network analysis perspective. J Psychiatr Res. (2021) 144:8–13. doi: 10.1016/j.jpsychires.2021.09.041, PMID: 34592511 PMC8665006

[41] Galderisi S Rucci P Kirkpatrick B Mucci A Gibertoni D Rocca P. Interplay among psychopathologic variables, personal resources, context-related factors, and real-life functioning in individuals with schizophrenia: a network analysis. JAMA Psychiatry. (2018) 75:396–404. doi: 10.1001/jamapsychiatry.2017.4607, PMID: 29450447 PMC5875306

[42] Galderisi S Rucci P Mucci A Rossi A Rocca P Bertolino A. The interplay among psychopathology, personal resources, context-related factors and real-life functioning in schizophrenia: stability in relationships after 4 years and differences in network structure between recovered and non-recovered patients. World Psychiatry. (2020) 19:81–91. doi: 10.1002/wps.20700, PMID: 31922687 PMC6953544

[43] Hu HX Lau WYS Ma EPY Hung KSY Chen SY Cheng KS. The important role of motivation and pleasure deficits on social functioning in patients with schizophrenia: a network analysis. Schizophr Bull. (2022) 48:860–70. doi: 10.1093/schbul/sbac017, PMID: 35524755 PMC9212088

[44] van Borkulo C Boschloo L Borsboom D Penninx BW Waldorp LJ Schoevers RA. Association of Symptom Network Structure with the course of [corrected] depression. JAMA Psychiatry. (2015) 72:1219–26. doi: 10.1001/jamapsychiatry.2015.2079, PMID: 26561400

[45] Bryant RA Creamer M O'Donnell M Forbes D McFarlane AC Silove D. Acute and chronic posttraumatic stress symptoms in the emergence of posttraumatic stress disorder: a network analysis. JAMA Psychiatry. (2017) 74:135–42. doi: 10.1001/jamapsychiatry.2016.3470, PMID: 28002832

[46] Southward MW Cheavens JS. Identifying core deficits in a dimensional model of borderline personality disorder features: a network analysis. Clin Psychol Sci. (2018) 6:685–703. doi: 10.1177/2167702618769560, PMID: 30854263 PMC6402351

[47] Gijzen MWM Rasing SPA Creemers DHM Smit F Engels RCME De Beurs D. Suicide ideation as a symptom of adolescent depression. A network analysis. J Affect Disord. (2021) 278:68–77. doi: 10.1016/j.jad.2020.09.029, PMID: 32956963

[48] Strauss GP Esfahlani FZ Kirkpatrick B Allen DN Gold JM Visser KF. Network analysis reveals which negative symptom domains are Most central in schizophrenia vs bipolar disorder. Schizophr Bull. (2019) 45:1319–30. doi: 10.1093/schbul/sby168, PMID: 30649527 PMC6811832

[49] Liu J Ma H He YL Xie B Xu YF Tang HY. Mental health system in China: history, recent service reform and future challenges. World Psychiatry. (2011) 10:210–6. doi: 10.1002/j.2051-5545.2011.tb00059.x, PMID: 21991281 PMC3188776

[50] van Borkulo CD Borsboom D Epskamp S Blanken TF Boschloo L Schoevers RA. A new method for constructing networks from binary data. Sci Rep. (2014) 4:5918. doi: 10.1038/srep05918 25082149 PMC4118196

[51] Hevey D. Network analysis: a brief overview and tutorial. Health Psychol Behav Med. (2018) 6:301–28. doi: 10.1080/21642850.2018.1521283, PMID: 34040834 PMC8114409

[52] Epskamp S Fried EI. A tutorial on regularized partial correlation networks. Psychol Methods. (2018) 23:617–34. doi: 10.1037/met0000167, PMID: 29595293

[53] Rubinov M Sporns O. Complex network measures of brain connectivity: uses and interpretations. NeuroImage. (2010) 52:1059–69. doi: 10.1016/j.neuroimage.2009.10.003, PMID: 19819337

[54] Boccaletti S Latora V Moreno Y Chavez M Hwang DU. Complex networks: structure and dynamics. Phys Rep. (2006) 424:175–308. doi: 10.1016/j.physrep.2005.10.009

[55] Opsahl T Agneessens F Skvoretz J. Node centrality in weighted networks: generalizing degree and shortest paths. Soc Networks. (2010) 32:245–51. doi: 10.1016/j.socnet.2010.03.006

[56] van Borkulo CD van Bork R Boschloo L Kossakowski JJ Tio P Schoevers RA. Comparing network structures on three aspects: a permutation test. Psychol Methods. (2022) 28:1273–1285. doi: 10.1037/met0000476, PMID: 35404628

[57] Epskamp S Borsboom D Fried EI. Estimating psychological networks and their accuracy: a tutorial paper. Behav Res Methods. (2018) 50:195–212. doi: 10.3758/s13428-017-0862-1, PMID: 28342071 PMC5809547

[58] Epskamp S Cramer AOJ Waldorp LJ Schmittmann VD Borsboom D. Qgraph: network visualizations of relationships in psychometric data. J Stat Softw. (2012) 48:1–18. doi: 10.18637/jss.v048.i04

[59] Christensen A Massara GP. Networktoolbox: methods and measures for brain, cognitive, and psychometric network analysis. R J. (2018) 10:422–39. doi: 10.32614/RJ-2018-065

[60] Shafer A Dazzi F. Meta-analysis of the positive and negative syndrome scale (Panss) factor structure. J Psychiatr Res. (2019) 115:113–20. doi: 10.1016/j.jpsychires.2019.05.008, PMID: 31128501

[61] Chang WC Wong CSM Or PCF Chu AOK Hui CLM Chan SKW. Inter-relationships among psychopathology, premorbid adjustment, cognition and psychosocial functioning in first-episode psychosis: a network analysis approach. Psychol Med. (2020) 50:2019–27. doi: 10.1017/S0033291719002113, PMID: 31451127

[62] Moura BM van Rooijen G Schirmbeck F Wigman H Madeira L Harten PV. A network of psychopathological, cognitive, and motor symptoms in schizophrenia Spectrum disorders. Schizophr Bull. (2021) 47:915–26. doi: 10.1093/schbul/sbab002, PMID: 33533401 PMC8266645

[63] Bellantuono L Marzano L La Rocca M Duncan D Lombardi A Maggipinto T. Predicting brain age with complex networks: from adolescence to adulthood. NeuroImage. (2021) 225:117458. doi: 10.1016/j.neuroimage.2020.117458, PMID: 33099008

[64] Jo YT Joo SW Shon SH Kim H Kim Y Lee J. Diagnosing schizophrenia with network analysis and a machine learning method. Int J Methods Psychiatr Res. (2020) 29:e1818. doi: 10.1002/mpr.1818, PMID: 32022360 PMC7051840

[65] Kelley SW Gillan CM. Using language in social media posts to study the network dynamics of depression longitudinally. Nat Commun. (2022) 13:870. doi: 10.1038/s41467-022-28513-3 35169166 PMC8847554

[66] Wang H Zhu R Dai Z Tian S Shao J Wang X. Aberrant functional connectivity and graph properties in bipolar ii disorder with suicide attempts. J Affect Disord. (2020) 275:202–9. doi: 10.1016/j.jad.2020.07.016, PMID: 32734909

[67] Kang R Wu Y Li Z Jiang J Gao Q Yu Y. Effect of community-based social skills training and tai-chi exercise on outcomes in patients with chronic schizophrenia: a randomized, one-year study. Psychopathology. (2016) 49:345–55. doi: 10.1159/000448195, PMID: 27584836

[68] Boschloo L van Borkulo CD Borsboom D Schoevers RA. A prospective study on how symptoms in a network predict the onset of depression. Psychother Psychosom. (2016) 85:183–4. doi: 10.1159/000442001, PMID: 27043457

[69] Bringmann LF Elmer T Epskamp S Krause RW Schoch D Wichers M. What do centrality measures measure in psychological networks? J Abnorm Psychol. (2019) 128:892–903. doi: 10.1037/abn0000446, PMID: 31318245

[70] McNally RJ. Can network analysis transform psychopathology? Behav Res Ther. (2016) 86:95–104. doi: 10.1016/j.brat.2016.06.006, PMID: 27424882

[71] Frost BG Tirupati S Johnston S Turrell M Lewin TJ Sly KA. An integrated recovery-oriented model (Irm) for mental health services: evolution and challenges. BMC Psychiatry. (2017) 17:22. doi: 10.1186/s12888-016-1164-3 28095811 PMC5240195

[72] Segarra R Ojeda N Peña J García J Rodriguez-Morales A Ruiz I. Longitudinal changes of insight in first episode psychosis and its relation to clinical symptoms, treatment adherence and global functioning: one-year follow-up from the Eiffel study. Eur Psychiatry. (2012) 27:43–9. doi: 10.1016/j.eurpsy.2010.06.003, PMID: 20813506

[73] Tominaga T Tomotake M Takeda T Ueoka Y Tanaka T Watanabe SY. Relationship between social and cognitive functions in people with schizophrenia. Neuropsychiatr Dis Treat. (2018) 14:2215–24. doi: 10.2147/ndt.S171207 30214211 PMC6121750

