# Supplementary material for: Violent behavior and the network properties of psychopathological symptoms and real-life functioning in patients with schizophrenia
Source: Front Psychiatry. 2024 Jan 11;14:1324911. doi: 10.3389/fpsyt.2023.1324911 (PMC10808501; doi:10.3389/fpsyt.2023.1324911)
Supplement: Supplementary file 1 [file Data_Sheet_1.PDF]

Supplemental Table 1 Evaluation criteria of psychopathological symptom and real-life functioning

**Item**	**Referenced scale**	**Evaluation Criteria**
Hallucinatory behavior	PANSS item P3	0=Absent/Questionable pathology (similar to PANSS item score≤2)<br>1=Present symptom(similar to PANSS item score≥3)
Suspiciousness and persecution	PANSS item P6	0=Absent/Questionable pathology (similar to PANSS item score≤2)<br>1=Present symptom(similar to PANSS item score≥3)
Excitement	PANSS item P4	0=Absent/Questionable pathology (similar to PANSS item score≤2)<br>1=Present symptom(similar to PANSS item score≥3)
Lack of spontaneity and flow of conversation	PANSS item N6	0=Absent/Questionable pathology (similar to PANSS item score≤2)<br>1=Present symptom(similar to PANSS item score≥3)
Passive apathetic social withdrawal	PANSS item N4	0=Absent/Questionable pathology (similar to PANSS item score≤2)<br>1=Present symptom(similar to PANSS item score≥3)
Mannerisms and posturing	PANSS item G5	0=Absent/Questionable pathology (similar to PANSS item score≤2)<br>1=Present symptom(similar to PANSS item score≥3)
Depression	PANSS item G6	0=Absent/Questionable pathology (similar to PANSS item score≤2)<br>1=Present symptom(similar to PANSS item score≥3)
Wandering	-	0=Absent<br>1=Run away from home without reason and purpose
Preoccupation	PANSS item G15	0=Absent/Questionable pathology (similar to PANSS item score≤2)<br>1=Present symptom(similar to PANSS item score≥3)
Affective lability	PANSS item S3	0=Absent/Questionable pathology (similar to PANSS item score≤2)<br>1=Present symptom(similar to PANSS item score≥3)
Aggression	MOAS	0=no aggression/verbal aggression/self-aggression (similar to MOAS subscore=0 in aggression against objects and aggression against others)<br>1=Aggression against objects/others(similar to MOAS subscore＞0 in aggression against objects and aggression against others)
Sleeping	-	0=Needs help or totally dependent<br>1=Totally self-sufficient
Eating	SLOF item 7.Eating	0=Needs help or totally dependent (similar to SLOF item score＜5)<br>1=Totally self-sufficient (similar to SLOF item score=5)
Personal care skills	SLOF domain “Personal care skills”	0=Needs help or totally dependent (similar to SLOF domain score＜35)<br>1=Totally self-sufficient (similar to SLOF domain score=35)

Supplementary Table 1 (continued)

Item	Referenced scale	Evaluation Criteria
Household management	SLOF item 27.Household responsibilities	0=Needs help or totally dependent (similar to SLOF item score＜5)<br>1=Totally self-sufficient (similar to SLOF item score=5)
Work skills	SLOF domain "Work skills"	0=Not highly typical of this person (similar to SLOF domain score＜30)<br>1=Highly typical of this person (similar to SLOF domain score=30)
Study skills	-	• Is able to learn new things<br>• Interest in the surroundings and news<br>0=Not highly typical of this person in any of the above points<br>1=Highly typical of this person in both of the above points
Interpersonal relationships	SLOF domain "Interpersonal relationships"	0=Not highly typical of this person (similar to SLOF domain score＜35)<br>1=Highly typical of this person (similar to SLOF domain score=35)

Note: PANSS, The Positive and Negative Syndrome Scale; MOAS, Modified Overt Aggression Scale; SLOF, Specific Level of Functioning Scale; -, no referenced scale.

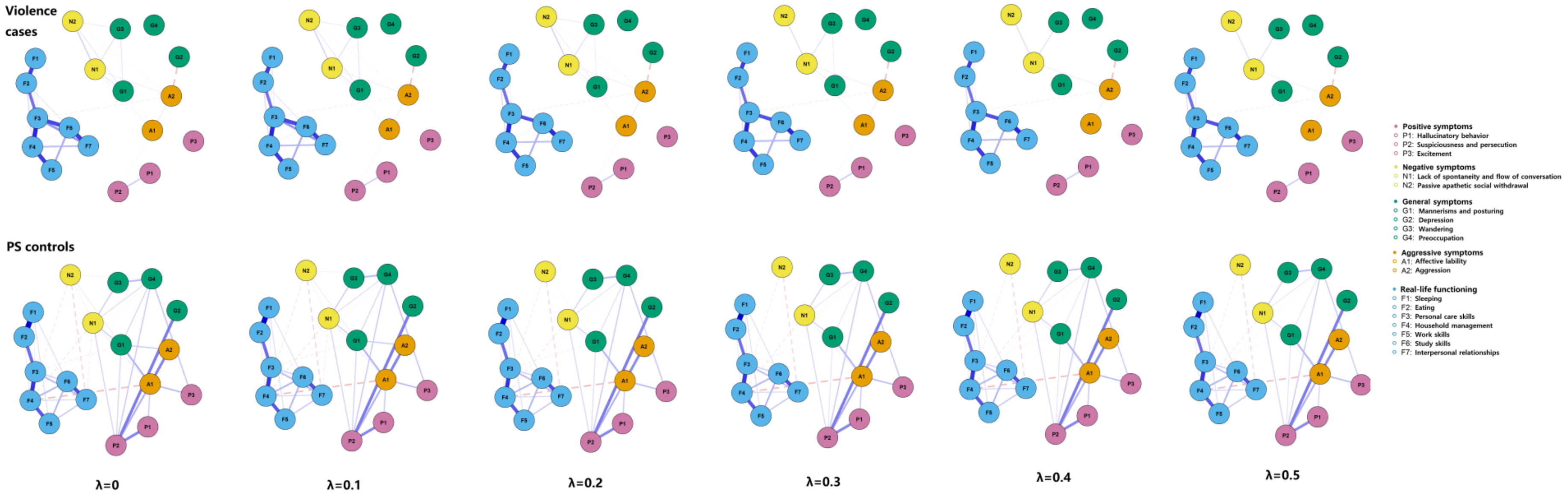

Supplementary Figure 1 Concentration networks of psychopathological symptoms and real-life functioning

γ, hyperparameter of EBIC; PS, Propensity score–matched; Solid edges indicate positive relationships, and dashed edges are negative relationships. The thickness of an edge represents the magnitude of the relationship.

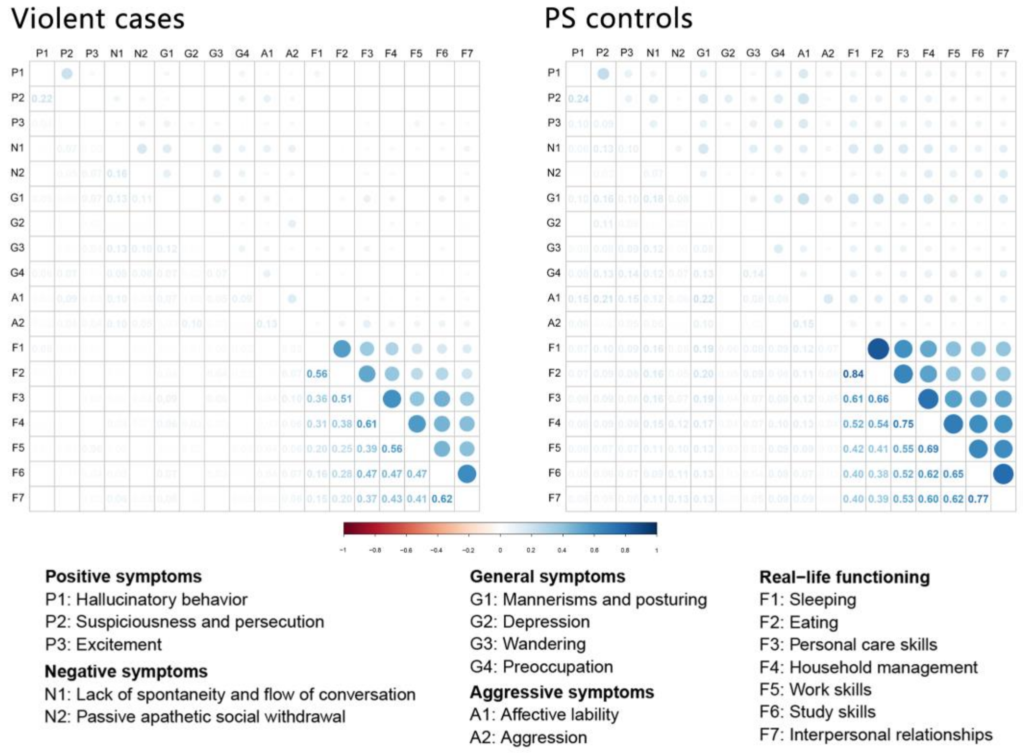

Supplementary Figure 2 Phi coefficients of psychopathological symptoms and real-life functioning PS, Propensity score–matched.

Supplementary Table 2 Network properties and comparisons with different hyperparameter γ

Network properties	Violence cases	Propensity score–matched controls	Propensity score–matched controls vs Violence cases	
			Test statistic	*P*
		**γ=0**		
Network structure	NA	NA	2.151	0.007*
Global network strength	30.022	45.912	15.890	0.012*
Network density	18.95%	28.76%	9.81%	0.646
CC	0.494	0.572	0.078	0.460
ASPL	2.331	2.007	-0.324	0.133
		**γ=0.1**		
Network structure	NA	NA	2.151	0.003*
Global network strength	30.022	45.912	15.890	0.015*
Network density	18.95%	28.76%	9.81%	0.694
CC	0.494	0.572	0.078	0.522
ASPL	2.331	2.007	-0.324	0.144
		**γ=0.2**		
Network structure	NA	NA	2.151	0.002*
Global network strength	27.647	43.225	15.577	0.028*
Network density	16.99%	26.14%	9.15%	0.777
CC	0.500	0.492	-0.008	0.965
ASPL	2.434	2.085	-0.349	0.108
		**γ=0.3**		
Network structure	NA	NA	2.151	0.002*
Global network strength	27.700	42.641	15.942	0.026*
Network density	15.03%	25.49%	10.46%	0.698
CC	0.444	0.539	0.094	0.458
ASPL	2.515	2.111	c-0.404	0.069
		**γ=0.4**		
Network structure	NA	NA	2.151	0.001*
Global network strength	25.665	42.401	16.735	0.016*
Network density	12.42%	25.49%	13.07%	0.544
CC	0.294	0.539	0.244	0.019*
ASPL	1.561	2.111	0.550	0.017*

Note: γ, hyperparameter; NA, not applicable; CC, global clustering coefficient; ASPL, global average shortest path length; * significant permutation test.

Supplementary Table 3 Adjacency matrixes of concentration networks

	P1	P2	P3	N1	N2	G1	G2	G3	G4	A1	A2	F1	F2	F3	F4	F5	F6	F7
**Violence cases**																		
**P1**																		
**P2**	0.83																	
**P3**																		
**N1**																		
**N2**				0.57														
**G1**																		
**G2**																		
**G3**				0.48														
**G4**																		
**A1**																		
**A2**				0.27			-0.97			0.24								
**F1**																		
**F2**												3.12						
**F3**											-0.30	0.30	2.25					
**F4**												0.33	0.59	3.49				
**F5**															3.16			
**F6**														2.79	0.21	1.27		
**F7**															1.08		3.41	
**Propensity score–matched controls**																		
**P1**																		
**P2**	1.93																	
**P3**																		
**N1**		0.63																
**N2**																		

Supplementary Table 3 (continued)

	**P1**	**P2**	**P3**	**N1**	**N2**	**G1**	**G2**	**G3**	**G4**	**A1**	**A2**	**F1**	**F2**	**F3**	**F4**	**F5**	**F6**	**F7**
**G1**		0.48		0.64														
**G2**		2.15																
**G3**				0.73														
**G4**		0.77	0.82	0.40		0.41		1.07										
**A1**	0.71	1.28	1.08	0.24		1.18												
**A2**										1.87								
**F1**																		
**F2**												4.19						
**F3**						-0.20						0.32	2.38					
**F4**				-0.40	-0.36				-0.15	-1.04		0.16	0.34	3.30				
**F5**												0.53			2.74			
**F6**												0.51		0.77	1.16	1.19		
**F7**					-0.64									1.07	0.63	0.88	2.88	

Note: P1, Hallucinatory behavior; P2, Suspiciousness and persecution; P3, Excitement; N1, Lack of spontaneity and flow of conversation; N2, Passive apathetic social withdrawal; G1, Mannerisms and posturing; G2, Depression; G3, Wandering; G4, Preoccupation; A1, Affective lability; A2, Aggression; F1, Sleeping; F2, Eating; F3, Personal care skills; F4, Household management; F5, Work skills; F6, Study skills; F7, Interpersonal relationships.

Supplementary Table 4 The CS-coefficients for centrality indices with different hyperparameter γ

Group	Strength	Betweenness	Closeness
	**γ=0**		
Violence cases	0.750	0.128	0
Propensity score–matched controls	0.750	0.206	0.05
	**γ=0.1**		
Violence cases	0.750	0.128	0
Propensity score–matched controls	0.672	0.128	0.05
	**γ=0.2**		
Violence cases	0.750	0.050	0
Propensity score–matched controls	0.750	0.128	0
	**γ=0.3**		
Violence cases	0.750	0.050	0
Propensity score–matched controls	0.750	0.050	0
	**γ=0.4**		
Violence cases	0.750	0	0
Propensity score–matched controls	0.750	0.050	0

Supplementary Table 5 Demographic and clinical characteristics in schizophrenia patients (sensitivity analysis)

**Characteristics**	**Schizophrenia patients (*N*=1667)**
Sex	
Male, *n* (%)	1117 (67.0)
Female, *n* (%)	550 (33.0)
Age(years), mean±*SD*	43.8±13.4
Age at onset(years), mean±*SD*	27.8±12.0
Duration of illness(years), mean±SD	16.1±10.5

Supplementary Table 6 Network variables in three follow-up assessments (sensitivity analysis)

**Variables**	**Schizophrenia patients (*N*=1667)**			***P***	
	**T-6, n (%)**	**T-3, n (%)**	**T, n (%)**	**T-3 vs T**	**T-6 vs T**
Hallucinatory behavior, *n* (%)	85 (5.1)	92 (5.5)	354 (21.2)	<0.001*	<0.001*
Suspiciousness and persecution, *n* (%)	122 (7.3)	131 (7.9)	347 (20.8)	<0.001*	<0.001*
Excitement, *n* (%)	69 (4.1)	82 (4.9)	215 (12.9)	<0.001*	<0.001*
Lack of spontaneity and flow of conversation, *n* (%)	213 (12.8)	224 (13.4)	396 (23.8)	<0.001*	<0.001*
Passive apathetic social withdrawal, *n* (%)	231 (13.9)	242 (14.5)	211 (12.7)	0.058	0.242
Mannerisms and posturing, *n* (%)	267 (16.0)	283 (17.0)	665 (39.9)	<0.001*	<0.001*
Depression, *n* (%)	13 (0.8)	11 (0.7)	31 (1.9)	0.002*	0.005*
Wandering, *n* (%)	43 (2.6)	41 (2.5)	161 (9.7)	<0.001*	<0.001*
Preoccupation, *n* (%)	135 (8.1)	147 (8.8)	260 (15.6)	<0.001*	<0.001*
Affective lability, *n* (%)	188 (11.3)	198 (11.9)	681 (40.9)	<0.001*	<0.001*
Aggression, *n* (%)	44 (2.6)	53 (3.2)	825 (49.5)	<0.001*	<0.001*
Sleeping, *n* (%)	701 (42.1)	693 (41.6)	170 (10.2)	<0.001*	<0.001*
Eating, *n* (%)	785 (47.1)	797 (47.8)	335 (20.1)	<0.001*	<0.001*
Personal care skills, *n* (%)	554 (33.2)	555 (33.3)	183 (11.0)	<0.001*	<0.001*
Household management, *n* (%)	394 (23.6)	377 (22.6)	83 (5.0)	<0.001*	<0.001*
Work skills, *n* (%)	206 (12.4)	208 (12.5)	35 (2.1)	<0.001*	<0.001*
Study skills, *n* (%)	225 (13.5)	217 (13.0)	49 (2.9)	<0.001*	<0.001*
Interpersonal relationships, *n* (%)	208 (12.5)	203 (12.2)	27 (1.6)	<0.001*	<0.001*

Note: * Statistically Chi-square test with Bonferroni's correction with α=0.025.

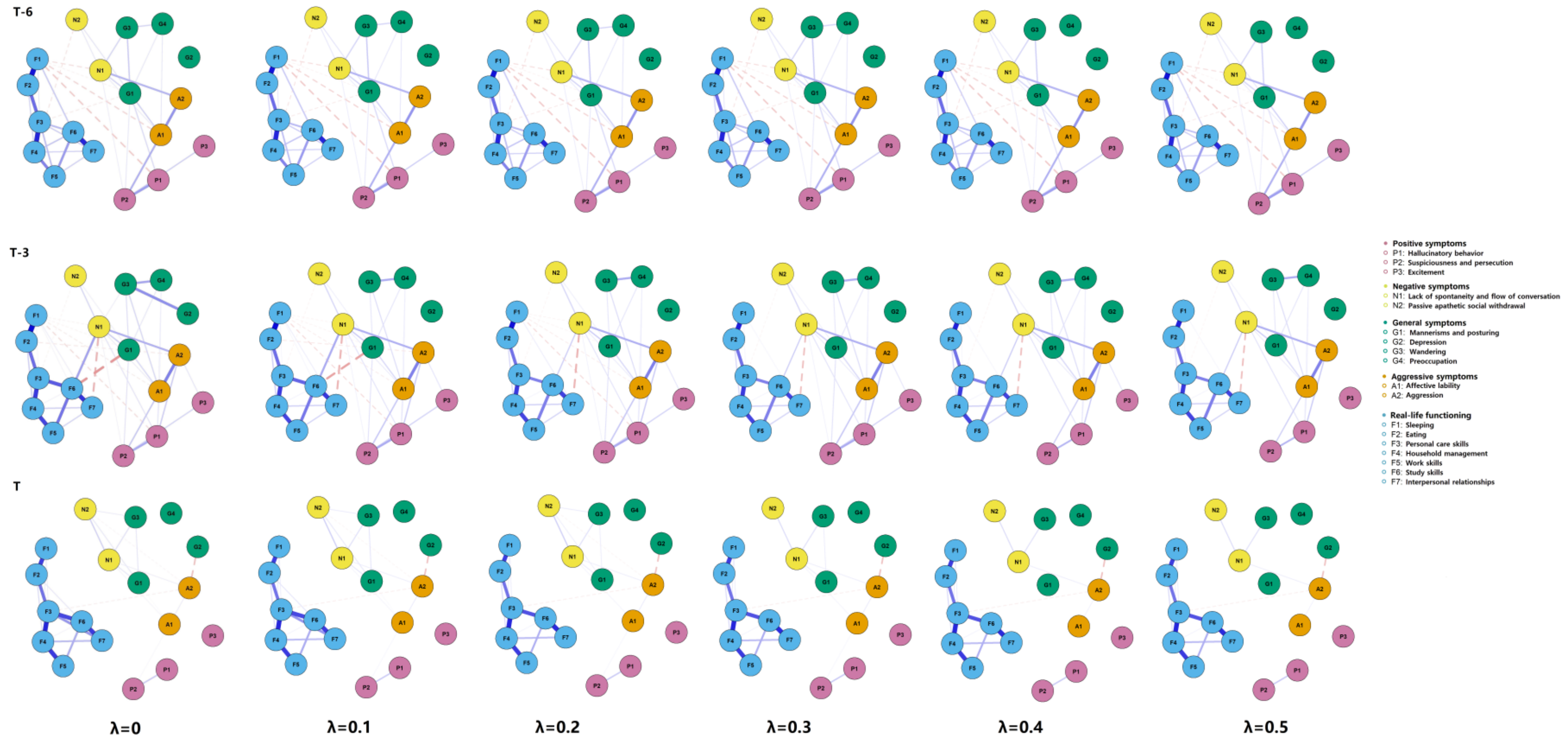

Supplementary Figure 3 Different concentration networks estimated with hyperparameter γ of EBIC (sensitivity analysis)

T, the first follow-up assessment after the first violence record; T-3, three-month before T follow-up assessment, T-6, six-month before T follow-up assessment. Solid edges indicate positive relationships, and dashed edges are negative relationships. The thickness of an edge represents the magnitude of the relationship.

Supplementary Table 7 Network properties and comparisons in four follow-up assessments with different hyperparameter γ (sensitivity analysis)

Network properties	T-6	T-3	T	T-6 vs T		T-3 vs T	
				Test statistic	*P*	Test statistic	*P*
			**γ=0**				
Network structure	NA	NA	NA	1.827	0.172	1.801	0.100
Global network strength	40.303	47.261	30.034	10.269	0.007*	17.227	< 0.001*
Network density	25.49%	30.72%	18.95%	6.54%	0.158	11.77%	0.024*
CC	0.531	0.438	0.494	-0.037	0.708	0.057	0.574
ASPL	1.945	1.994	2.331	0.386	0.053	0.337	0.112
			**γ=0.1**				
Network structure	NA	NA	NA	1.827	0.123	1.690	0.113
Global network strength	40.303	44.170	30.034	10.269	0.004*	14.136	< 0.001*
Network density	25.49%	29.41%	18.95%	6.54%	0.137	10.46%	0.043
CC	0.531	0.464	0.494	0.037	0.734	-0.031	0.789
ASPL	1.945	1.779	2.331	-0.386	0.071	-0.552	0.016*
			**γ=0.2**				
Network structure	NA	NA	NA	1.670	0.205	1.690	0.096
Global network strength	40.303	40.637	27.656	12.648	< 0.001*	12.982	< 0.001*
Network density	25.49%	27.45%	16.99%	8.50%	0.048	10.46%	0.031
CC	0.531	0.479	0.500	0.031	0.752	-0.021	0.846
ASPL	1.945	1.903	2.434	-0.489	0.033	-0.531	0.027
			**γ=0.3**				
Network structure	NA	NA	NA	1.670	0.192	1.690	0.085
Global network strength	40.303	38.834	26.710	13.593	0.001*	12.124	0.001*
Network density	25.49%	25.49%	15.30%	10.19%	0.015*	10.19%	0.021
CC	0.531	0.528	0.444	0.087	0.429	0.084	0.507
ASPL	1.945	2.083	2.515	-0.570	0.037	-0.432	0.122
			**γ=0.4**				
Network structure	NA	NA	NA	1.776	0.107	1.778	0.046
Global network strength	38.121	37.147	25.670	12.450	0.001*	11.477	0.002*
Network density	23.53%	23.53%	12.42%	11.11%	0.006*	11.11%	0.012*
CC	0.521	0.545	0.294	0.227	0.046	0.251	0.040
ASPL	1.809	2.173	1.561	0.248	0.350	0.612	0.044
			**γ=0.5**				
Network structure	NA	NA	NA	1.776	0.093	1.778	0.040
Global network strength	38.121	36.567	25.670	12.450	< 0.001*	10.896	0.001*
Network density	23.53%	22.88%	12.42%	11.11%	0.001*	10.46%	0.012*
CC	0.521	0.573	0.294	0.227	0.036	0.279	0.031
ASPL	1.809	1.949	1.561	0.248	0.407	0.388	0.219

Note: T, the first follow-up assessment after the first violence record; T-3, three-month before T follow-up assessment, T-6, six-month before T follow-up assessment; NA, not applicable; * statistically significant permutation test with Bonferroni's correction with α=0.025; CC, global clustering coefficient; ASPL, global average shortest path length.

Supplementary Table 8 The CS-coefficients for centrality indices in three follow-up assessments with different hyperparameter γ(sensitivity analysis)

Group	Strength	Betweenness	Closeness
		**γ=0**	
T-6	0.750	0.128	0
T-3	0.672	0	0
T	0.750	0.050	0
		**γ=0.1**	
T-6	0.750	0.128	0
T-3	0.750	0	0
T	0.750	0.050	0
		**γ=0.2**	
T-6	0.750	0.128	0
T-3	0.672	0	0
T	0.750	0.050	0
		**γ=0.3**	
T-6	0.750	0.050	0
T-3	0.672	0	0
T	0.750	0.050	0
		**γ=0.4**	
T-6	0.750	0.05	0
T-3	0.750	0	0
T	0.750	0	0
		**γ=0.5**	
T-6	0.750	0	0
T-3	0.750	0	0
T	0.750	0	0

Note: T, the first follow-up assessment after the first violence record; T-3, three-month before T follow-up assessment, T-6, six-month before T follow-up assessment.

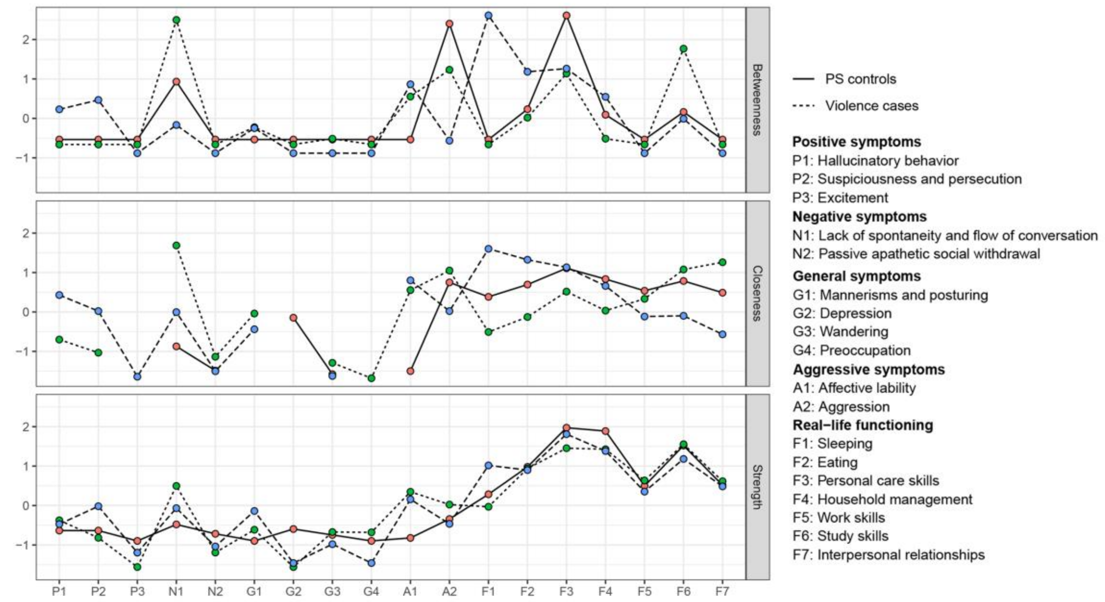

Supplementary Figure 4 The standardized centrality measures of networks in three follow-up assessments (sensitivity analysis)

Closeness is missing because some nodes are isolate in post-violence network.

T, the first follow-up assessment after the first violence record; T-3, three-month before T follow-up assessment, T-6, six-month before T follow-up assessment.
